# Analysis of the metals and metalloids concentrations and of the bacterial population in sediments of the Red River Delta, Vietnam

**DOI:** 10.3389/fmicb.2024.1394998

**Published:** 2024-06-05

**Authors:** Sandrine Chifflet, Thuoc Chu van, Vuong Bui Van, Thu Pham The, Xavier Mari, Nathalie Pradel

**Affiliations:** ^1^Aix Marseille Université, IRD, Université de Toulon, CNRS, MIO, UM 110, Marseille, France; ^2^Institute of Marine Environment and Resources (IMER), Vietnam Academy of Science and Technology (VAST), Haiphong, Vietnam

**Keywords:** bacterial population, metals and metalloids, Red River Delta, bioremediation, diversity

## Abstract

In this study, we discuss for the first time the relationships between the diversity of the bacterial population and of the metals and metalloids concentrations in the sediments of the Red River Delta, Vietnam. The analysis of the 16S rRNA by the Illumina technology revealed a diversified population and a potential of bioremediation by the microorganisms, notably by the *Bacilli* relatively abundant in the Bach Dang estuary, where high metals and metalloids concentrations were highlighted. This work offers new information on the environmental context of the delta and highlights the potential impact that metals and metalloids may have on the bacterial population. Further investigations on the role of the bacteria in the biogeochemistry of this ecosystem will be of interest for the development of bioremediation processes.

## 1 Introduction

The estuarine and coastal areas of the Red River Delta (Northern Vietnam) are bounded by Halong and Haiphong cities (northeast and southwest of the delta, respectively), and by Cat Ba Island (Southeast of the delta). Halong Bay is a UNESCO World Heritage Site since 1994,^1^ located to the north of the delta. It covers an area of 1,500 km^2^ and includes 1969 limestone karst islets. The bay attracts every year more than 2.5 million tourists and is controlled by the blue economy (e.g., aquaculture, fishing, shipping). However, anthropogenic sources of contamination are numerous (mining activities, metallurgical industries, waste incineration, agricultural practices, transport, fossil fuel combustion, etc.) and can threaten microbial diversity (Rochelle-Newall et al., 2011; Chu et al., 2014). The Red River’s name is derived from its reddish-brown waters, indicating a considerable sediment load (1,500 to 7,000 m^3^.s^−1^), rich in iron oxides (Le et al., 2007, 2022). Due to high organic particulate content, approximately 50% of the metals and metalloids (MM) fluxes drained by the Red River are deposited in sediments (Le et al., 2022; Roberts et al., 2022). MM spatial distributions have been found mainly dependent of the organic matter and clay minerals from the Red River catchment (Ho et al., 2010). Among the consequences of these inputs, high concentrations of MM in the delta may damage DNA and may disrupt the function of proteins and the enzymatic activities of organisms (Balali-Mood et al., 2021; Witkowska et al., 2021; Mitra et al., 2022). In Halong Bay, concentrations of Cu (14.5 g g^−1^), Pb (30.4 g g^−1^) and As (6.1 g g^−1^) in nearshore sediments are above sediment quality guidelines and decrease offshore toward the Gulf of Tonkin (Hoai et al., 2020).

It is well known that microorganisms’ communities are highly sensitive to environmental parameters and therefore are considered indicators of local environmental conditions (Blakely et al., 2002; Schloter et al., 2003). They may also reduce the levels of pollutants. It has been hypothesized that indigenous microbial communities that have become adapted to long-term MM inputs play a key role in the remediation of metal-rich ecosystems (Chen et al., 2018). However, no study regarding the bacterial communities inhabiting the Red River Delta has been performed until now. The closest marine locations studied for microbial diversity have been reported in the Asian Sea, at more than 300 km to the East of the Gulf of Tonkin (Zhu et al., 2013; Niu et al., 2017; Cui et al., 2019; Yuan et al., 2019; Zhang et al., 2019).

In this study, we investigated the MM concentrations and the diversity of bacteria by 16S rRNA gene V4 region Illumina analysis in the marine sediments of the Red River Delta, to give further insights on their distribution and their role in the bioremediation processes in the delta.

## 2 Materials and methods

### 2.1 Study sites and sediments sampling

The Red River (1,150 km long) originates from the mountainous region of Yunnan in Southern China (2000 m above the sea level) and drains 169,000 km^2^ of catchment area (48.8% in China, 50.3% in Vietnam and 0.9% in Laos). The river takes its name from the reddish-brown colour of its waters due to its high sediment load rich in iron dioxide, which enter in Haiphong Bay, directly connected to Halong Bay (Northeastern Vietnam). The Ha Long Bay is a semi-enclosed bay located in the Gulf of Tonkin. It is a tectonically active region, which has allowed the formation of numerous islets that characterize the Halong Bay. Some of these islets have sedimentary lakes submitted to atmospheric deposition, like on the Hang Trai islet. The Haiphong and Halong Bay are part of the Red River Delta (14,300 km^2^), the discharge plain of the Red River and its main tributaries, such as the Bach Dang river.

**Figure 1:**
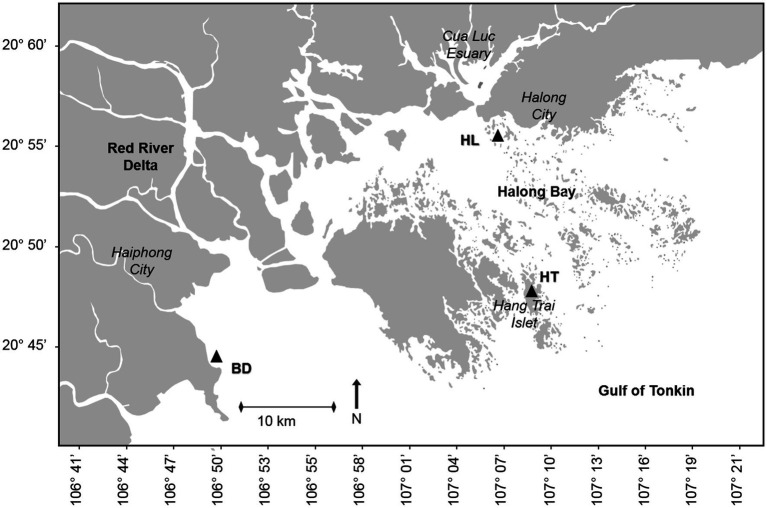
Location of the sampling stations HL, HT and BD, in the Red River Delta of the Gulf of Tonkin (black triangles). HL, site located in the coastal area of Halong city; HT, site located in a lacustrine lake in Hang Trai islet; BD, site located in the mangrove area of the Bach Dang estuary.

Samples were collected in the Red River Delta in September 2019. Three sampling stations, named HL (for Halong Bay), HT (for Hang Trai Islet), and BD (for Bach Dang estuary), were selected based on various anthropogenic inputs and natural forcing (Figure 1). HL (20°55′6.04”N; 107°6′30.81″E) is in the coastal area near Halong city. This station is impacted by local urban and industrial runoff, diffuse atmospheric deposition and terrigenous inputs from the Red River. HT (20°47′4.85”N; 107°7′27.88″E) is in a salty enclosed lake on the Hang Trai Islet. It is impacted by diffuse atmospheric deposition. BD (20°45′28.39”N; 106°46′22.91″E) is in the Bach Dang Estuary, influenced by the terrigenous inputs of the Red River as well as anthropogenic inputs from the watershed.For each station, a coastal sediment core (between 1 to 5 meters below the water surface) was handly collected with a piston sediment corer equipped with a plexiglass tube (100 cm long, 60 mm inner diameter). HL, HT and BD cores measured 81, 66 and 57 cm, respectively. The cores were stored at 4°C up the laboratory. Sediment cores were then gradually extruded from the plexiglass tubes using a piston and sliced with a ceramic knife into consecutive increments under sterile conditions: 1 cm from 0 to 20 cm depth, 2 cm between 20 and 30 cm depth, and 3 cm from 30 cm to the bottom. The samples (whole slices) were then sealed in plastic bags and frozen until to analyses. They were used for bacterial diversity and MM analyses.

### 2.2 Metals and metalloids analyses

Sample processing was carried out in a clean laboratory (ISO 5) at the MIO Marine Chemistry Laboratory (Aix Marseille University), using bi-distilled (HCl and HNO_3_) and commercial grade (HF; Optima grade, Fisher Chemical) acids as well as ultrapure water (Milli-Q Integral 3, Millipore). The perfluoroalkoxy vials (PFA vials, Savillex) were pre-cleaned in consecutive acid baths (HCl 10%, 90°C, 24 h; HNO_3_ 10%, 90°C, 24 h). The analytical tubes (metal-free centrifuge tubes, VWR) were pre-cleaned in HCl 10% overnight at room temperature. PFA vials and analytical tubes were thoroughly rinsed then dried under a laminar flow hood (ISO 1) before using. Samples (about 100 mg of dw sediment) were digested in PFA vials in a mixture of pure acids (HNO_3_/HCl/HF, 4/4/1 v/v), heated on a hot-block (120°C, 24 h), evaporated to near dryness, and re-dissolved into 3 mL of HNO_3_ (2%). The digested samples were then diluted in analytical tubes using HNO_3_ (2%) before running MM analyses.

MM analyses (As, Cd, Cr, Cu, Ni, Fe, Pb, Sb, Zn) were performed using an inductively coupled plasma mass spectrometer (ICP-MS NexION 300; Perkin Elmer). Procedural blanks were below detection limits and considered as negligible. Quality controls were performed using a certified marine sediment reference material (MESS-4). Recovery results ranged between 88 and 101%, depending on the element.

### 2.3 Bacterial 16S rRNA gene sequencing

DNA of microorganisms was extracted from 400 μg of each consecutive sample slice along the sediment cores using the GenElute Soil DNA extraction kit (Sigma-Aldrich). The extracted and purified DNA was resuspended in 60 μL pure H_2_O, at a final concentration > 20 μg/μl, and then used as template for the sequencing at MR DNA (www.mrdnalab.com, Shallowater, TX, United States). The 16S rRNA gene V4 variable region PCR primers 515/806 were used in a 30 cycles PCR using the HotStarTaq Plus Master Mix Kit (Qiagen, United States) under the following conditions: 95°C for 5 min, followed by 30–35 cycles of 95°C for 30 s, 53°C for 40 s and 72°C for 1 min, after which a final elongation step at 72°C for 10 min was performed. After amplification, PCR products were checked in 2% agarose gel to determine the success of amplification and the relative intensity of bands. The samples were multiplexed using unique dual indices and were pooled together in equal proportions based on their molecular weight and DNA concentrations. Pooled samples were purified using calibrated Ampure XP beads. Then the pooled and purified PCR product were used to prepare an Illumina DNA library. Forward and reverse sequencing was performed for each sample slice, at MR DNA (www.mrdnalab.com, Shallowater, TX, United States) on a Mi-Seq following the manufacturer’s guidelines.

### 2.4 16S rRNA data processing

Sequence data were processed using MR DNA analysis pipeline (MR DNA, Shallowater, TX, USA). The Q25 sequence data derived from the sequencing process was processed using the MR DNA ribosomal and functional gene analysis pipeline (www.mrdnalab.com, MR DNA, Shallowater, TX, United States). Sequences were depleted of primers, short sequences <150 bp and sequences with ambiguous base calls were removed. Sequences were quality filtered using a maximum expected error threshold of 1.0 and dereplicated. The dereplicated or unique sequences were denoised; unique sequences identified with sequencing or PCR point errors were removed, followed by chimera removal, thereby providing a denoised sequence or zOTU. OTUs were defined clustering at 3% divergence (97% similarity). Final zOTUs were taxonomically classified using BLASTn against a curated database derived from NCBI^2^ and compiled into each taxonomic level by both counts of the number of sequences and by the relative (proportion) percentage of sequences within each sample that map to the designated taxonomic classification.

### 2.5 Statistical analyses

All statistical analyses were performed using XLSTAT 2023.1.4 (Microsoft Excel add-in program; Addinsoft, Paris, France). Student tests were used to determine the significant differences between samples or according to the sediment depth, regarding the bacterial diversity and the MM concentrations. Differences with *p* values <0.05 were considered statistically significant. Analyses of variance (ANOVA) followed by Tukey’s pairwise comparison tests were used to assess differences in the dominant bacterial classes (> 1% of the relative abundance) or MM concentrations between core levels or sites. Redundancy analysis (RDA) was used to examine the effect of MM concentrations (explanatory matrix) on bacterial diversity (from classes/orders, response matrix). Differences with *p* values <0.05 were considered statistically significant. Shannon index was used for the alpha-diversity estimation; beta-diversity was evaluated using Sorensen index.

### 2.6 Nucleotide sequences accession numbers

MiSeq V4 amplicons raw sequence data were deposited in the Sequence Read Archive (SRA) of NCBI under BioProject accession number PRJNA1017876.

## 3 Results and discussion

### 3.1 Overview of the bacterial communities in the Red River Delta

**Figure 2:**
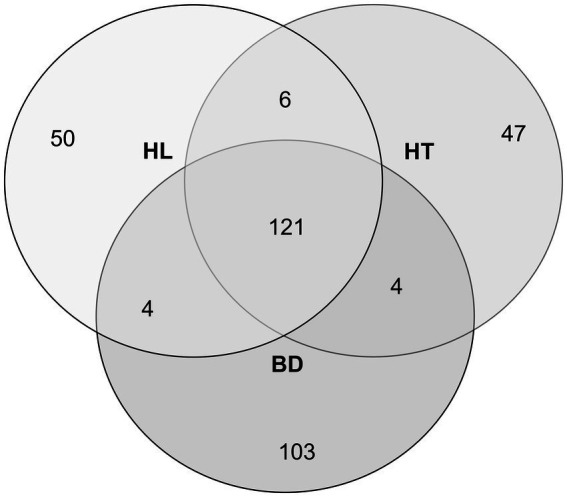
Venn diagram of the different bacterial genera number, according to the sampling stations HL, HT, and BD.

The diversity and structure of the bacterial communities were investigated using Illumina high-throughput sequencing technology to sequence the 16S rRNA gene V4 variable region from sediments samples collected at three sites of the Red River Delta (HL, HT, and BD). After filtering the low-quality reads and trimming the adapters and barcodes, there were 255,976, 255,462, and 189,159 effective sequences of bacteria generated from the samples collected at HL, HT, and BD, respectively (BioProject accession number PRJNA1017876). The sequences were assigned to 678, 630, and 784 OTUs, respectively. Overall, the taxonomic assignations revealed that they were affiliated within 232 genera (181, 177, 171 genera in HL, HT, BD, respectively), within 69 orders within 34 classes. Venn diagram shows unique and common shared genera at the three sites (Figure 2). The great majority of the genera (121, more than 70%) were found in common at the three sites (Sorensen indexes of 0.71, 0.71, and 0.72 for HL vs. BD, HL vs. HT, and HT vs. BD, respectively). However, the richness diversity (alpha-diversity) of the sediment samples were found different, with the richness at HL and HT being higher (Shannon indexes of 4.32 and 3.40, respectively), than at BD (Shannon index of 2.26). Overall, the Shannon indexes values suggest equilibrated ecosystems, notably at HL and HT.

**Figure 3:**
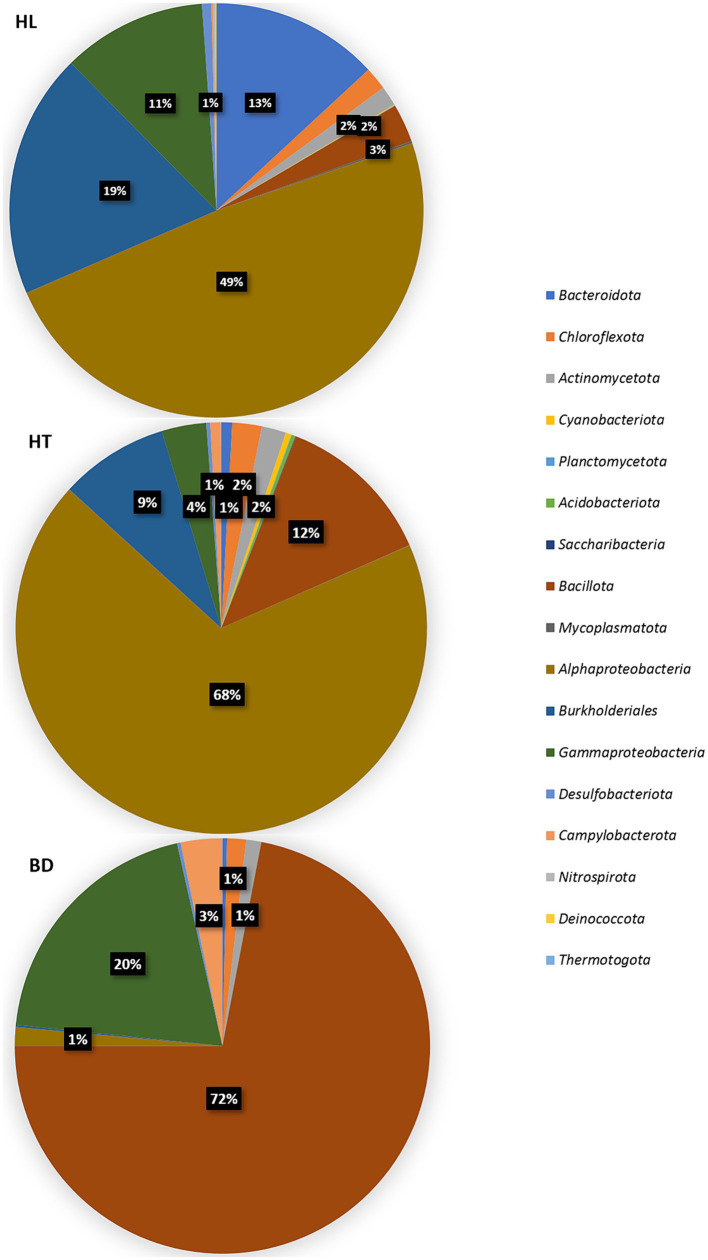
Distribution of 16S (V4 region) sequences (% reads number) at the phylum, class, or order levels, according to each station.

The distribution of the bacterial sequences reads (% relative to the bacterial reads number) are shown at the phylum level, except the *Pseudomonadota* (*Proteobacteria*) shown by classes/orders, in Figure 3. Almost all the phyla are represented in the sediments (with a majority of *Pseudomonadota*, *Bacillota* (*Firmicutes*), *Bacteroidota* (*Bacteroidetes*), *Chloroflexota* (*Chloroflexi*) and *Actinomycetota* (*Actinobacteria*)), suggesting diverse sources of carbon and energy in these ecosystems. The BD station was distinct compared to the HL and HT stations in the relative abundance of the dominant bacterial phylotypes (ANOVA tests followed by Tukey’s multiple comparison tests, *p* < 0.05). Significant differences were found for the dominant phylotypes *Alphaproteobacteria* (*Pseudomonadota*), *Campylobacterota, Bacillota* and for the order *Burkholderiales* (*Pseudomonadota*). The major important point resides in the dominance of *Bacillota* at BD (72% relative abundance, mostly composed of *Bacilli*), whereas *Alphaproteobacteria* were dominant at the HL and HT stations (49 and 68% respectively). In addition, the order *Burkholderiales* was present in higher proportion at HL and HT than at BD (19, 9%, and less than 1%, respectively), and *Campylobacterota* (genus *Sulfurovum*) were more abundant at BD (3% versus less than 1% at HL and HT).

These results suggest different sources of carbon and energy at the three sites, or different sources of contaminants (urban and industrial at HL, atmospheric deposition at HT, terrigenous and anthropogenic inputs at BD), which may be responsible of the occurrence of different proportions of microorganisms in these area.

### 3.2 Analysis of the bacterial population according to the sediments’ depth

**Figure 4:**
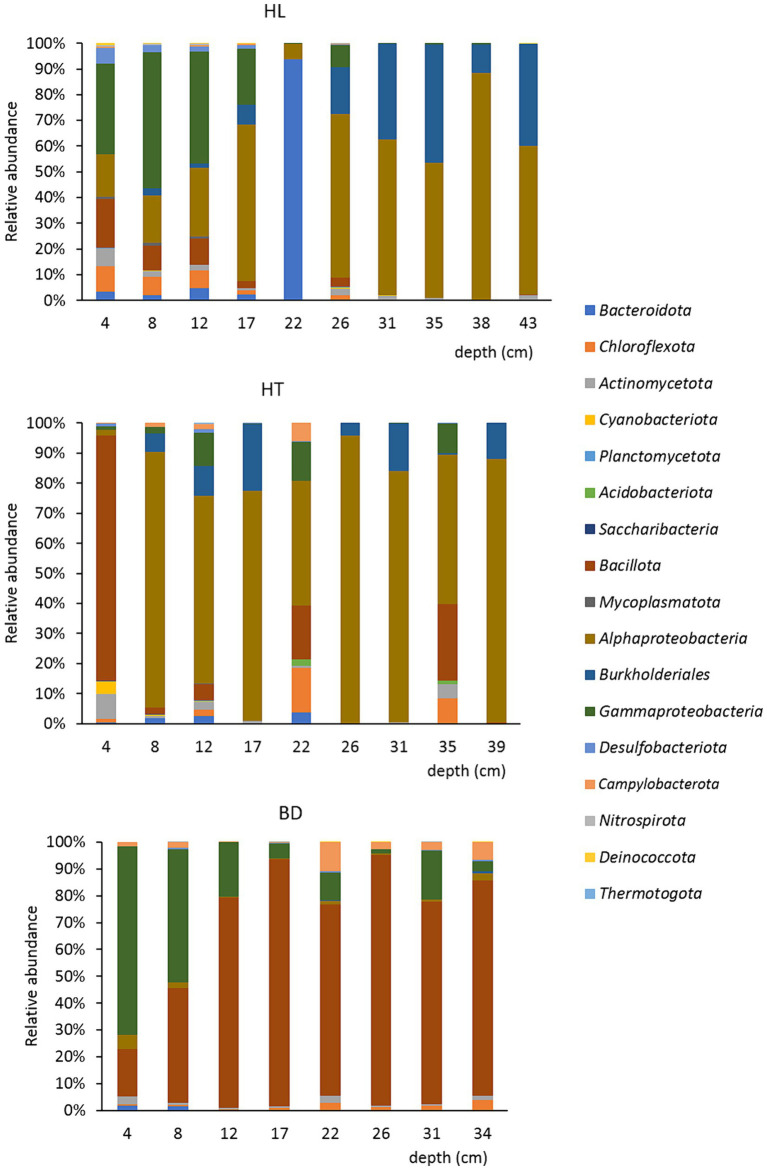
Repartition of the bacterial communities at the phylum/class/order levels, at each station, according to the depth (cm).

Regarding the bacterial surface and bottom populations of the sediment’s samples (upper or below 20 cm depth), the most notable difference was observed at the site HL (Figure 4). Significant differences were found between the bacterial surface and bottom populations (ANOVA tests followed by Tukey’s multiple comparison tests, *p* < 0.05). The Shannon alpha-diversity index was higher for the surface (5.19) than for the bottom (3.24) samples. Moreover, the Sorensen beta-diversity index was low between the upper and depth layers (0.47), indicating a stratification of the sediments at this site by comparison to HT and BD, where no clear difference of bacterial population was evidenced according to the depth. For example, the species *Candidatus* Magnetobacterium (phylum *Nitrospirota* (*Nitrospirae*)), found in the first 20 cm of the HL sediments, is microaerophile and is found in stratified environments (Lin et al., 2014). It is to notice, too, the abundance of the microaerobic *Salinimicrobium* genus (phylum *Bacteroidota*) around 20 cm depth. Significant differences were found between the first 20 cm and the bottom for the *Gammaproteobacteria*, *Bacteroidota*, *Desulfobacterota* (higher proportions at the surface) and for the *Burkholderiales* (higher proportion at the bottom). Overall, it suggests the presence of an oxic-anoxic transition zone (OATZ) around 20 cm depth in the sediments at the HL station. In addition, bacteria involved in the biogeochemical cycles of iron and sulfur have been evidenced in the first 20 cm of the core HL sample, such as *Sulfurimonas* sp. (sulfur-oxidizing, *Campylobacterota*), *Leptothrix* sp. (Fe^II^-oxidizing, *Burkholderiales*), and *Ferrimonas* sp. (Fe^III^-reducing genus, *Gammaproteobacteria*). Therefore, it can be hypothesized that oxygen is available for microorganisms in the first centimetres in HL sediments, whereas more anaerobic conditions may occur at HT and BD, where strict anaerobes were found in the first centimeters, as for example *Desulfotomaculum halophilum* (sulfur-reducing, *Bacillota*) at the BD site. This could be due to surface current occurring at HL, whereas backwaters conditions would be present at HT and BD. Regarding HT, it is to highlight that the first five centimeters of sediments contained a majority of *Planococcus citreus* (56%; belongs to the class *Bacilli*, phylum *Bacillota*). This species is known to degrade complex extracellular proteins, such as those of shrimps (Alvarez, 1982). The presence of this species may be due to chitin and keratin from shellfishes and fishes, which may deposit to the bottom of the lake of the Hang Trai Islet.

### 3.3 Comparison of the bacterial communities at the genus level between the three sites

Among the 181 bacterial genera detected at HL, most of the sequences (> 10,000 reads) corresponded to those of *Sphingomonas* (22.1%; *Alphaproteobacteria*), *Agrobacterium* (14.9%; *Alphaproteobacteria*), *Salinimicrobium* (12.3%; *Bacteroidota*), *Delftia* (7.5%; *Burkholderiales*), and *Bordetella* (7.4%; *Burkholderiales*). Interestingly, *Sphingomonas* spp. degrade aromatic compounds and are thus of interest for environmental depollution processes (Seo et al., 2009). This genus has been found in metal rich areas (Sultana et al., 2023). The genus *Salinimicrobium* degrade recalcitrant organic matter and is able to resist to MM using efflux pumps systems (Rezaei Somee et al., 2018). Among the 177 genera found at the HT, *Agrobacterium* (51.1%), *Planococcus* (6.7%; *Bacilli*), and *Sphingomonas* (4.4%) were mostly detected (> 10,000 reads). Interestingly, the genus *Dehalococcoïdes* (phylum *Chloroflexota*) and the order *Burkholderiales* (phylum *Pseudomonadota*), mainly found in HL and HT cores (around 3% of the total genera, and 19 and 9% of the total orders from HL and HT, respectively) are able to degrade aromatic hydrocarbons (Matturro et al., 2023).

Among the 171 bacterial genera of the BD site, the most represented were *Bacillus* (64.2%; *Bacilli*) and *Vibrio* (14%; *Gammaproteobacteria*). *Bacillus* spp. can produce endospores that allow them to resist adverse environmental conditions (Nicholson et al., 2000). In addition, this genus has been widely studied for its properties to resist and adapt to MM contaminations, due to its Gram-positive membrane properties (Wróbel et al., 2023). The *Vibrio* genus is ubiquitous and has also been documented as being resistant to high MM concentrations (Su et al., 2022). It is to be noticed that genus *Microbulbifer*, belonging to the *Gammaproteobacteria*, is well represented at BD (2% of the total genera), and has been reported to degrade complex carbohydrates and aromatic compounds (González et al., 1997).

Overall, most anaerobic heterotrophs micro-organisms have been evidenced at the three sites. A majority of microorganisms able to degrade complex organic matter were found at HL, and a majority of *Bacillus* spp. at BD, known to adapt to high MM contaminations. These data suggest different and efficient bioremediation potentials in the bacterial communities of the sediments in the Red River Delta, according to the contamination sources. Complex and recalcitrant organic matter, such as aromatic hydrocarbons, may govern the presence of organisms able to degrade these compounds at HL, whereas high MM concentrations may govern the presence microorganisms tolerant and resistant to heavy metals at BD.

### 3.4 Metals and metalloids concentrations in the Red River Delta

MM concentrations measured in the sediments are presented in the Table 1. The range of MM concentrations found at HL, HT and BD was consistent with the values generally measured in sediments of the Halong Bay, which includes the Cua Luc Estuary, the Red River Delta, and the mangrove area near Haiphong City (Tue et al., 2012; Ho et al., 2013; Nguyen et al., 2016).

**Table 1:** MM concentrations in the Gulf of Tonkin and sediment quality guidelines.

	Fe	As	Cd	Cr	Cu	Ni	Pb	Sb	Zn	
	mg/g	μg/g	μg/g	μg/g	μg/g	μg/g	μg/g	μg/g	μg/g	
**Sediments in Halong Bay**										
HL core - Mean	26.8	12.2	0.06	56.1	18.0	21.0	24.8	1.49	67.9	This study
Min	21.4	9.7	0.04	41.9	11.5	15.0	18.7	1.18	47.9	This study
Max	33.2	15.0	0.09	67.7	140.2	26.0	31.4	1.95	155.0	This study
HT core - Mean	26.0	33.2	2.37	127.1	23.8	57.3	27.3	1.44	193.5	This study
Min	20.6	17.5	1.76	96.7	17.4	45.4	20.7	0.80	152.0	This study
Max	34.2	53.6	4.05	164.9	37.4	74.2	37.3	2.08	260.2	This study
BD core - Mean	56.9	42.5	0.22	116.4	67.1	58.0	87.7	3.71	162.0	This study
Min	50.8	31.3	0.17	105.9	56.1	52.9	71.4	3.00	140.5	This study
Max	67.8	57.9	0.27	125.6	77.2	63.9	98.4	4.57	175.9	This study
Mangrove Haiphong	NA	NA	0.44	68.6	77.4	NA	80.2	NA	132.3	Tue et al. (2012)
Cua Luc Estuary	18.9	NA	NA	44.0	14.0	19.0	18.0	NA	65.0	Ho et al. (2013)
Red River Delta	40.7	NA	0.18	102.3	63.0	44.0	71.0	NA	144.0	Nguyen et al. (2016)
**Sediment quality guidelines**										
ERL	NA	8.2	1.2	81.0	34.0	20.9	46.7	2	150.0	Long et al. (1995)
ERM	NA	70.0	9.6	370.0	270.0	51.6	218.0	25	410.0	Long et al. (1995)
TEL	NA	7.2	0.7	52.3	18.7	15.9	30.2	NA	124.0	MacDonald et al. (1996)
PEL	NA	41.6	4.2	160	108.0	42.8	112.0	NA	271.0	MacDonald et al. (1996)

NA, Not Available; ERL, Effect Range Low; ERM, Effect Range Medium; TEL, Threshold Effect Level; PEL, Probable Effect Level.

Overall, higher MM concentrations were found in HT and BD cores than in the HL core, with the highest As, Cu, Ni and Pb concentrations at BD (mean 42.5 μg.g^−1^, 67.1 μg.g^−1^, 58.0 μg.g^−1^ and 87.7 μg.g^−1^, respectively), and the highest Cd and Sb concentrations at HT (mean 2.37 μg.g^−1^ and 3.71 μg.g^−1^, respectively). These results confirm distinct biogeochemical environments between sites of the Red river Delta. The Sediment Quality Guidelines (SQG) propose empirical indexes for assessing biological effects in contaminated sediments (Long et al., 1995; MacDonald et al., 1996). The indexes ERL (Effect Range Low) or TEL (Threshold Effect Level) correspond to MM concentrations below which no harmful effects on organisms are likely to occur, and the indexes ERM (Effect Range Medium) or PEL (Probable Effect Level) represent concentrations above which harmful effects are likely to occur. In this study, MM concentrations at HL were mainly below the ERL and TEL values, while those at HT and BD were between the low and high SQG values, with Ni concentrations at BD above the ERM and PEL thresholds. These results indicate that there may be moderate ecotoxicological risks for benthic organisms in the Red River Delta, with lower risk at HL and HT than at BD.

### 3.5 Relationships between bacterial phylotypes and metals and metalloids concentrations

**Figure 5:**
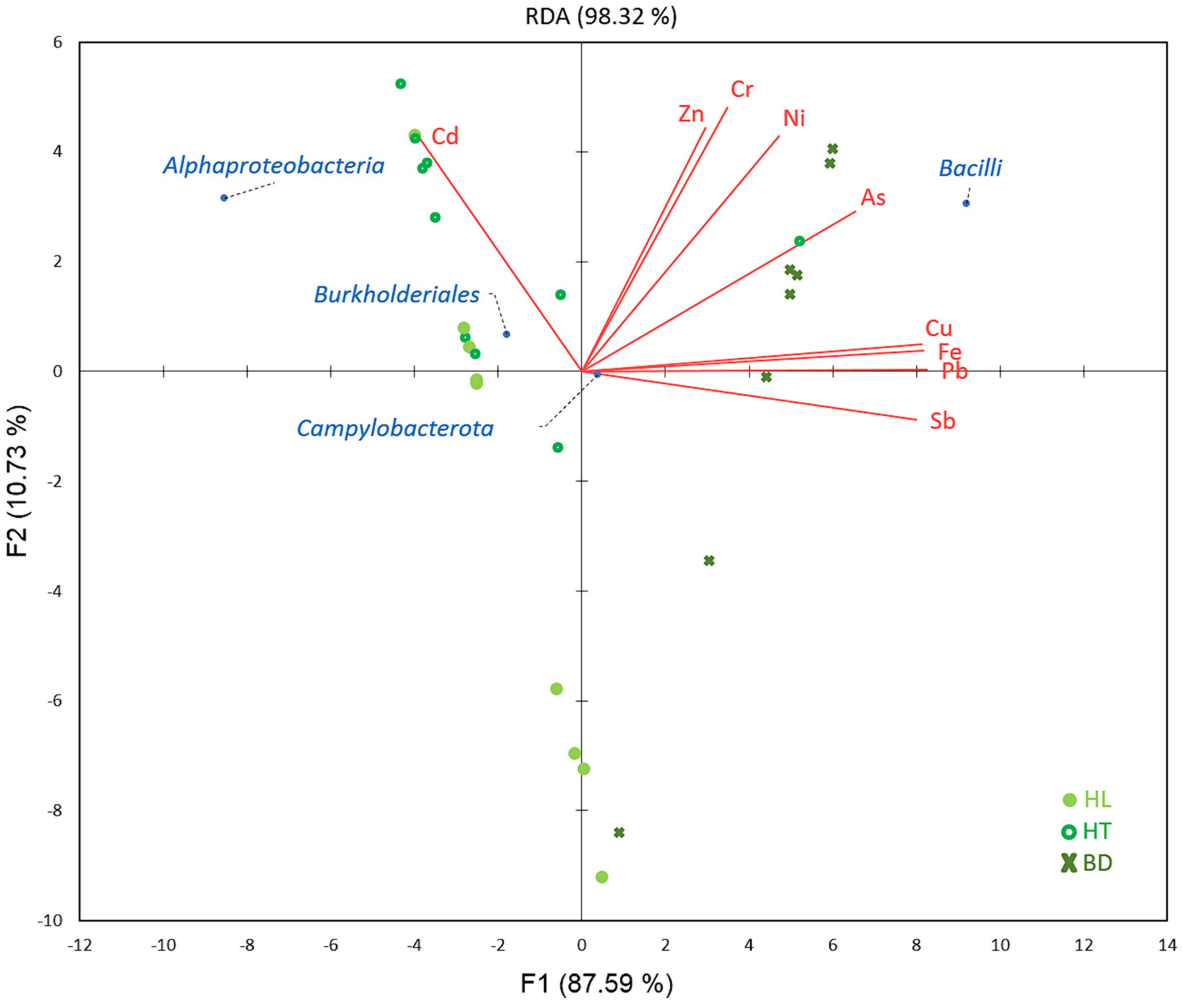
Relations between MM concentrations and bacterial classes/orders, according to the stations of the Red River Delta.

Redundancy analysis (RDA) was used to study the relationships between the significant different dominant phylotypes between the three sites (phylum *Campylobacterota,* classes *Alphaproteobacteria* and *Bacilli* and order *Burkholderiales*) and MM concentrations in sediments, according to the sampling sites (Figure 5). Results showed that *Alphaproteobacteria* and *Burkholderiales* were significantly correlated with Cd at HL and HT (*p* < 0.05), while *Bacilli* were correlated with As, Cr, Cu, Fe, Ni, Pb, Sb, and Zn at BD (p < 0.05).

The link between the presence of Gram-positive *Bacilli* and the highest MM concentrations at the BD site, notably As and Ni,may indicate an adaptation of the bacterial community to this environment. Most *Bacilli* belonged to the genus *Bacillus* (64.2%), reported to be able to adsorb metals to its cell surface, and to resist and adapt to the highest MM concentrations (Wróbel et al., 2023). A recent study has shown that contamination with multiple MM decreases microbial diversity and favours generalists as the keystones in microbial occurrence networks (Qi et al., 2022). The lowest value of diversity index obtained at BD vs. HL and HT sites comfort this idea and suggest that the genus *Bacillus* may play a role as generalist in this ecosystem rich in MM. It is to notice that the genus *Sulfurovum* (phylum *Campylobacterota*), a sulfur oxidizing bacterium reducing nitrate and reported to mobilize MM (Ihara et al., 2017), was linked to MM and to the BD station, too (ANOVA test, *p* < 0.05).

In addition, bacteria previously evidenced as able to resist and remove MM were found in all three cores (without significant differences between sites, so not represented in the RDA analysis). Among them, the Gram-positive genus *Micrococcus* (phylum *Actinomycetota*) has been reported to remove Ni and Pb by biosorption (Igiri et al., 2018), and the genus *Pseudomonas* (class *Gammaproteobacteria*, order *Alteromonadales*) resists, reduces, and removes several metals (Ni, Cu, Cd, Pb, Cr) by the way of efflux pumps (Igiri et al., 2018). This genus was often isolated from long-term MM contaminated area (Chen et al., 2018). Finally, a high relative abundance of class *Clostridia*, known to resist to Ni (Igiri et al., 2018), was found at the three stations, too. It should be noticed that recent works report the isolation, from the Bach Dang Estuary (BD site), of a new species, *Tepidibacter aestuarii*, belonging to the *Clostridia*, able to resist to high Ni concentrations (Fouteau et al., 2022; Pradel et al., 2024).

## 4 Conclusion

Studies on prokaryotic communities have received limited attention in coastal waters of the Asian areas, despite the presence of ecological threats caused by anthropogenic impacts, such as intensive agricultural and industrial activities, as well as international shipping and tourism development. In addition, no data have been previously reported to date on the relationships between microbial communities and MM concentrations in sediments from the Gulf of Tonkin. This study is the first investigation of microbial diversity in the Red River Delta. Analysis of the microbial diversity associated with the sediment samples constitutes the first step for their bioprospection in bioremediation processes.

Regarding the values of the diversity indexes, the bacterial communities seem equilibrated whatever the site of the Red River Delta. The values were like that reported in other Asian seas areas studies (Zhu et al., 2013). Therefore, it can be hypothesized that no recent major contamination events occurred at the Red River Delta and that an efficient equilibrium of the bacterial communities occurred in these ecosystems. It suggests the presence of microorganisms which may have developed tolerance and resistance capacities to the contaminants. Heterotrophs were present in majority in the three stations, with *Pseudomonadota, Bacillota, Bacteroidota, Chloroflexota*, and *Actinomycetota* accounting for the largest proportions of the identified phyla. Previous studies have indicated the dominance of these phyla in metal- and organic matter-rich environments (Chen et al., 2018). *Bacteroidota* ferment complex organic matter, with the capacity to degrade polysaccharides (Acosta-González et al., 2013; Cui et al., 2019). *Chloroflexota* and *Actinomycetota* contribute to aromatic hydrocarbons degradation (Fry et al., 2008; Pöritz et al., 2015; Cui et al., 2019). *Actinomycetota*, being Gram-positive microorganisms, may play an important role in bioremediation by biosorption of MM, too.

Differences in the distribution of several taxa were highlighted, which may be controlled by the different organic matter sources and MM concentrations in the ecosystems of the Red River Delta. *Alphaproteobacteria* was the dominant class at HL (coastal) and HT (lacustrine) sites. They have been frequently found in metal- and complex organic matter-rich environments (Chen et al., 2018). Among the *Pseudomonadota*, the order *Burkholderiales,* which presents the potential for aromatic compounds catabolism (Pérez-Pantoja et al., 2012; Cui et al., 2019), is also highly abundant at HL and HT.

The BD site is in a mangrove area of the Red River Delta, which drains effluents from intensive agricultural activities and domestic/industrial wastewaters from the surrounding cities. *Bacillota* (notably *Bacilli*) and *Gammaproteobacteria* (notably *Vibrio*) were enriched at this station. Their abundance underlines a nutrient- and metal-rich area, probably caused by these human activities. The phylum *Bacillota* is largely composed of Gram-positive species, such as *Bacillus* spp. and *Clostridium* spp. with the potential to bind and sequester metals (Gavrilescu, 2004). They have high adsorptive capacities due to the peptidoglycan and teichoic acid content in their cell walls (Beveridge and Fyfe, 1985). *Gammaproteobacteria* play an important role in the degradation of organic compounds and oil-contaminants (Cui et al., 2019). It has been evidenced as the major class of bacteria found in the sediments of the South and East China Seas and has been reported as beneficial for detoxification of pollutants in mangrove sediments (Zhu et al., 2013; Gong et al., 2019; Zhang et al., 2019). Being metabolically versatile, this class is ecologically very successful.

Overall, a large part of the bacterial genera evidenced at the three sites HL, HT and BD are able to degrade recalcitrant and complex organic matter and to resist to MM.

Thus, these sediments constitute different and important sources of new microorganisms, which will be explored for their capacity in heavy metals adsorption or precipitation properties or in aromatic hydrocarbons degradation, for future use in biotechnological processes as the bioremediation of contaminated areas. These practices may be efficient, low cost and environmental-friendly.

For example, a significant correlation was found between high As and Ni concentrations and the presence of a high proportion of *Bacilli* in the sediments of the BD site. A focus on the isolation of new microorganisms from this site, as for example new *Clostridia* or *Bacilli*, will be performed. Their resistance systems to heavy metals will be studied at the molecular level to understand their remediation ability towards heavy metal ions. Genomic sequencing selected isolates should pave the way for the discovery of genes involved in heavy metal tolerance.

Moreover, this study depicts an overview of the bacterial communities at different places in the Red River Delta, to create a basis of study of the evolution of the effect of contaminants in the Gulf of Tonkin on the bacterial population over the time. A better knowledge of the microbial diversity is therefore essential for environmental quality assessment and should be further explored. The data obtained here will be compared to bacterial diversity analyses which will be obtained in future works. It will be performed at several places in the Red River Delta, and a long-term monitoring will be implemented. In addition, further research employing metagenomic and metaproteomic analyses will be required to further elucidate the response of microbial community to environmental stressors.

## Data availability statement

The datasets presented in this study can be found in online repositories. The names of the repository/repositories and accession number(s) can be found below: https://www.ncbi.nlm.nih.gov/, PRJNA1017876.

## Author contributions

SC: Conceptualization, Data curation, Formal analysis, Funding acquisition, Investigation, Methodology, Project administration, Software, Writing – original draft. TC: Investigation, Resources, Writing – original draft. VB: Methodology, Writing – original draft. TP: Investigation, Resources, Writing – original draft. XM: Conceptualization, Formal analysis, Funding acquisition, Investigation, Resources, Supervision, Validation, Visualization, Writing – original draft. NP: Conceptualization, Data curation, Formal analysis, Funding acquisition, Investigation, Methodology, Software, Supervision, Validation, Visualization, Writing – original draft, Writing – review & editing.

## Footnotes

^1^http://whc.unesco.org/fr/list/672/

^2^www.ncbi.nlm.nih.gov
